# Pericapsular Nerve Group (PENG) Block Versus Lumbar Erector Spinae Plane Block (ESPB) in Pediatric Hip Surgery: A Randomized, Double-Blinded, Controlled Trial

**DOI:** 10.1097/BPO.0000000000002882

**Published:** 2024-12-18

**Authors:** Malgorzata Reysner, Tomasz Reysner, Piotr Janusz, Grzegorz Kowalski, Milud Shadi, Przemysław Daroszewski, Katarzyna Wieczorowska-Tobis, Tomasz Kotwicki

**Affiliations:** Departments of * Palliative Medicine; † Spine Disorders and Pediatric Orthopedics, University of Medical Sciences; ‡ Department of Organization and Management in Health Care, Poznan University of Medical Sciences, Poznań, Poland

**Keywords:** PENG, ESPB, hip surgery, nerve block, regional anesthesia, pain management

## Abstract

**Background:**

The effectiveness and safety of the pericapsular nerve group (PENG) block and lumbar erector spinae plane block (ESPB) in pediatric hip surgeries is limited mainly to case reports. This study assessed the efficacy of ultrasound-guided PENG block versus lumbar ESPB under spinal anesthesia.

**Methods:**

Ninety patients aged 2 to 7 years, ASA I-III scheduled for hip surgery were randomly assigned to 3 equal groups, each receiving the PENG block group (n=30), the ESPB group (n=30), or the control group (n=30). After the spinal anesthesia, the block was performed with 0.5 kg/mL of 0.2% ropivacaine. The primary outcome was the pain scores (FLACC) 48 hours after surgery. The secondary outcomes included postoperative FLACC pain scores, neutrophile-to-lymphocyte ratio (NLR), platelet-to-lymphocyte ratio (PLR), and total opioid consumption.

**Results:**

The FLACC score was significantly lower in the lumbar ESPB and PENG groups compared with the control group (*P*<0.0001) at all time points. The NLR and PLR levels were substantially lower in the PENG and lumbar ESPB groups (*P*<0.0001) compared with the control group. The NLR and PLR levels were significantly lower in the PENG and lumbar ESPB groups compared with the control group (*P*<0.0001). The total opioid consumption was significantly lower in the PENG and lumbar ESPB groups compared with the control group (*P*<0.0001). Forty-three percent of children in the PENG group and 50% of children in the lumbar ESPB group did not require opioids postoperatively.

**Conclusions:**

The PENG block and the lumbar ESPB provide efficient postoperative analgesia in children undergoing hip surgery. The PENG block and lumbar ESPB lower cumulative opioid consumption and the stress response to surgery, expressed by NLR and PLR levels.

**Level of Evidence:**

Level I.

Pediatric hip surgery is frequently associated with significant pain in the postoperative period.^1^ Several regional analgesic techniques have been proposed to manage postoperative pain after pediatric hip surgery. However, femoral nerve blocks can be associated with difficulty in mobility in the immediate postoperative period. Hip surgery is associated with the risk of peripheral nerve injury,^2^ and prolonged sensory block can delay the recognition and treatment of nerve injury. In contrast, motor-sparing blocks like the pericapsular nerve group (PENG) and lumbar erector spine plane block (ESPB) can help avoid this complication.^3,4^ The PENG block and lumbar ESPB have been successfully used in adult hip surgeries.^5^ However, the evidence of PENG block and lumbar ESPB in children’s hip surgery is not well established.^6^

In addition to pain control, regional anesthesia can inhibit the stress response.^7^ The surgery and anesthesia activate the neuroendocrine system.^8^ Thus, it triggers the release of neuroendocrine hormones and cytokines. Postoperative pain, described as inflammatory, nociceptive, and neuropathic, is often associated with surgical stress response. The systemic alterations of leukocytes, including lymphopenia and neutrophilia leukocytosis, frequently arise in response to surgery.^9^ The platelet-to-lymphocyte ratio (PLR) and neutrophil-to-lymphocyte ratio (NLR) are often used as immune system markers against various noninfectious stimuli.^10^ The stress response is affected by the surgical trauma and the anesthetic method.^11^

We designed this randomized, controlled, double-blinded clinical trial to compare the effects of lumbar ESPB and PENG block on postoperative analgesia after a pediatric hip surgery. Our primary endpoint was pain scores 48 hours after surgery. The secondary endpoints included pain scores at other time points, total opioid consumption, and the NLR and PLR levels.

## METHODS

### Study Design

This double-blinded and prospective RCT was conducted in a single center in Poland. The Poznan University of Medical Sciences Bioethics Committee approved the study on March 9, 2023, protocol number 224/23. The trial was registered on July 5, 2023, at ClinicalTrials.gov (NCT06087549). Written informed consent was obtained from all patients’ caregivers for these scientific contributions. Enrollment occurred from October 17, 2023 to February 23, 2024. The study was conducted following the Helsinki Declaration.

### Participants

Enrollment was proposed before surgery to caregivers of children scheduled for elective unilateral periacetabular osteotomy due to hip dysplasia under spinal anesthesia, aged above 2 months and below 12 years old, and American Society of Anesthesiologists physical status (ASA) I, II, or III.

Patients were not included in this study if their caregivers refused to participate; had a history of chronic pain defined by the use of gabapentin/pregabalin for >3 months or opioid use >1 repeated opioid prescription in the last 3 months; had an infection of the site of the needle puncture; were <2 months old or were older than 12 years old, patients classified as ASA III were excluded due to the presence of other comorbidities, had known or suspected coagulopathy.

All participants underwent unilateral periacetabular osteotomy for hip dysplasia without additional procedures such as adductor tenotomy or capsular release. The surgical technique was standardized across all cases to ensure uniformity.

### Randomization and Concealment

Computer software randomly assigned patients 1:1:1 to receive ultrasound-guided PENG block, lumbar ESPB, or the control group, using a randomization list generated by the nQuery Advisor program (Statistical Solutions, Boston, MA).

The double-blinding in this treatment was accomplished through the strict design of the work tasks for the researchers, who were unaware of each other’s final scores. The first researcher, uninvolved in the study, prepared the randomization list and camouflaged the group assignments in closed, opaque, and serially numbered envelopes. The other consultant anesthesiologist tracked the administrators to open the envelopes before applying the PENG block or lumbar ESPB to reveal the group assignments. The separate anesthesiologist was responsible for performing the blocks, and steps were taken to ensure that other care providers remained blinded. As a result, the anesthesia team, surgeons, operating room staff, and patients were blinded to the study group assignment. The group blinding was unmasked after the statistical analysis was accomplished.

All patients underwent hip surgery under spinal anesthesia by one surgical team at the Orthopaedical Hospital at Poznan University of Medical Sciences.

The patients underwent at least 2 days of active follow-up after surgery. An independent researcher gathered the primary and secondary outcomes during in-patient hospital visits.

### Procedures

In all 3 groups, the patients received Midazolam 0.25 mg/kg p.o. a half hour before the surgery as a part of the multimodal preemptive analgesia protocol. All patients received standardized spinal anesthetic management under mild sedation as practical commonly in our hospital. Mild sedation was performed with continuous propofol infusion at 5 mg/kg/hour, which was continued throughout the entire surgery. Spontaneous ventilation was maintained with an oxygen mask at 2 L/min. Spinal anesthesia (L3/4, PAJUNK, sprotte needle 27 G, 70 mm) was performed with 0.1 mL/kg of 0.5% ropivacaine. There was no surgeon-delivered periarticular infiltration during surgery. Two anesthesiologists performed the blocks. All had at least 5 years of experience in postspecialty clinical expertise focused on pediatric regional anesthesia.

### PENG Block Procedure

**FIGURE 1:**
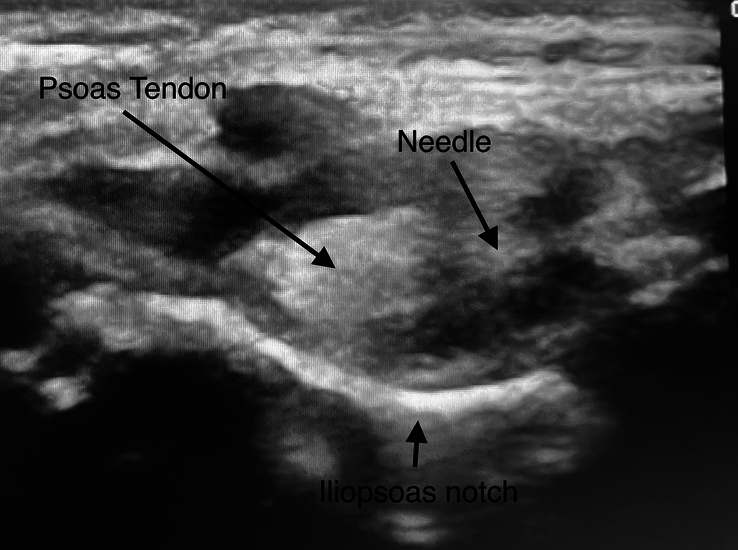
Ultrasound-guided PENG block. PENG indicates pericapsular nerve group.

After the spinal anesthesia and before the surgical incision, the PENG block was performed. The patient was placed in the supine position. We used a linear, high-frequency 4 to 8 MHz sonographic ultrasound probe and a 22-gauge needle (Stimuplex Ultra 360, 50 mm). The puncture was accomplished in the lateromedial direction. To avoid quadriceps weakness, the needle was placed away and more laterally of the iliopsoas tendon between the iliopubic eminence and anteroinferior iliac spine.^12–14^ Hydrolocation positioning was performed with 0.5 mL of 0.9% isotonic saline. After the negative aspiration, 0.5 mL/kg of 0.2% ropivacaine was placed laterally to the iliopsoas tendon (Fig. 1).

### Lumbar ESPB Procedure

**FIGURE 2:**
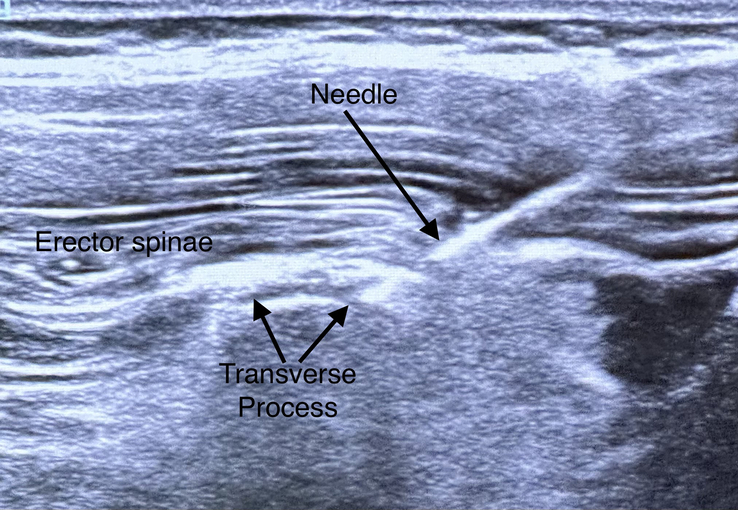
Ultrasound-guided ESPB. ESPB indicates erector spinae plane block.

After the spinal anesthesia and before the surgical incision, the ESPB was performed at the L3 vertebral level. The patient was placed in the lateral position with the operated side up. We used a linear, high-frequency 4 to 8 Hz probe, and a 22-gauge needle (Stimuplex Ultra 360, 50 mm) was used. The transducer was placed in a paramedian sagittal orientation at the level of spinous processes. The needle was inserted in-plane from cranial to caudal direction until the tip contacted the transverse process. We injected 0.5 mL of 0.9% isotonic saline to confirm the proper injection plane by visualizing the spread deep to the erector spinae muscles and superficial to the transverse process. After the negative aspiration, 0.5 mL/kg of 0.2% ropivacaine was placed to ensure the fascial plane between the transverse process and the erector spinae muscle (Fig. 2).

### Postoperative Analgesia Management

The postoperative analgesia included the administration of acetaminophen 15 mg/kg every 6 hours, metamizole 15 mg/kg every 6 hours, and ibuprofen 10 mg/kg every 8 hours. In addition, if the patient’s FLACC (Face, Legs, Activity, Cry, Controllability Scale) score was 4 or above, a 0.1 mg/kg nalbuphine bolus injection was administrated for rescue analgesia.

### Outcome Measures

#### Primary Outcome

At all postoperative time points (30, 60, 90, 120 min, 6, 12, 24, and 48 h after surgery), the pain score was assessed using the FLACC score (0 meaning no pain and 10 meaning the worst pain imaginable). The final score was agreed upon at the end of the examination set.

#### Secondary Outcomes

Two independent physicians evaluated the subject during the examination. The final score was agreed upon at the end of the examination. Also, the total opioid consumption was accessed from the postoperative and orthopaedic wards by the residents and fellows, who were blinded to the study. Blood samples for PLR and NLR were obtained 12 and 24 hours after surgery by nurses who were blinded to the study. Two researchers blinded to the group allocation assessed the outcomes.

### Statistical Analysis

The sample size was based on our primary hypothesis that adding L-ESPB or the PENG block to standard analgesia procedures improves pain management. Comparisons between the 3 groups are rare in the literature, especially regarding pediatric patients. Therefore, our sample size calculations were based on literature regarding adult patients. Tulgar et al^15^ compared the control group with 2 nerve blocks (PENG block and Quadratus Lumborum Group) and used the NRS to assess the pain. Upon examination of the study results, it was found that the average NRS value of the QLB group was 1.00±0.65, the ESPB group was 1.45±0.52, and the control group 3 hours after surgery was 2.00±0.46. The study’s effect size was 1.13 and was calculated based on these results. Evaluating the literature regarding PENG block^6,14,16,17^ and ESPB^16,18–20^ in adults and children, the average 24-hour pain score in the nerve block groups was found to be 2, which was similar to the results of the nerve block groups in the Tulgar and colleagues study. On the basis of the above calculations, this study sample size was calculated using G-power software (3.1.9.6; Institute of Experimental Psychology, Heinrich Heine University, Dusseldorf, Germany). The sample size was determined to be 26 per group, with Bonferroni correction, with a power of 0.95 and a significance level of 0.05. The 90 (30×3) children were recruited to facilitate block randomization and consider the dropouts.

Statistical analysis was performed using GraphPad Prism 10.1.1 (270) software (GraphPad Software Inc., San Diego, CA). The parametric distribution of numerical variables was evaluated using the Shapiro-Wilk normality test. The ANOVA test with post hoc Tukey test assessed differences between groups. Categorical variables were compared with Kruskal-Wallis test, and an analysis of contingency was compared with a Fisher exact test. A *P*-value <0.05 was considered statistically significant.

## RESULTS

### Summary of Participation

**FIGURE 3:**
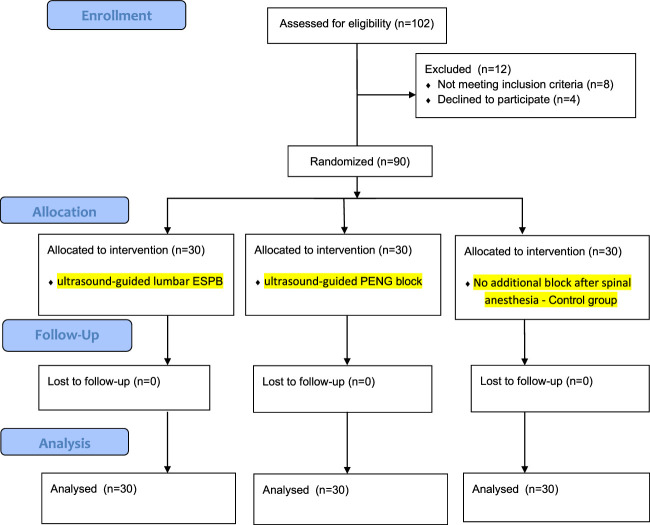
Consort 2010 flow diagram.

Of the 102 children assessed for eligibility, 8 did not meet the inclusion criteria, and 4 children’s caregivers refused to participate. The remaining 90 were randomly allocated to 3 groups and analyzed, as seen in Figure 3. No clinically relevant differences were apparent from group characteristics, as shown in Table 1.

**TABLE 1:** Baseline Characteristics

	Control group (n=30)	PENG block group (n=30)	Lumbar ESPB group (n=30)	*P* *, † (95% Cl of diff.)	*P* †, ‡ (95% Cl of diff.)	*P* †§ (95% Cl of diff.)	*P* †∥
Age (y)	5.5 (1.6)	5.1 (1.6)	4.8 (1.6)	0.66 (-0.6281 to 1.361)	0.25 (-0.3281 to 1.661)	0.75 (-0.6948 to 1.295)	0.28
Weight (kg)	20.2 (5.9)	19.1 (4.4)	17.2 (4.3)	0.63 (-1.870 to 4.203)	0.05 (-0.03674 to 6.037)	0.33 (-1.203 to 4.870)	0.07
Height (cm)	108.0 (16.5)	107.8 (9.8)	101.6 (13.2)	1.00 (-8.104 to 8.437)	0.17 (-1.937 to 14.60)	0.18 (-2.104 to 14.44)	0.12
ASA	2.3 (0.52)	2.4 (0.56)	2.4 (0.50)	0.74 (-0.4247 to 0.2247)	0.44 (-0.4914 to 0.1580)	0.88 (0.3914 to 0.2580)	0.47
Time of surgery (min)	120.0 (36.9)	142.0 (41.9)	132.2 (42.0)	0.09 (-46.84 to 2.839)	0.48 (-37.01 to 12.67)	0.61 (-15.01 to 34.67)	0.11

* *P*-value compares the control group to the PENG group.

† ANOVA test with post hoc Tukey test used to compare means between the groups.

‡ *P*-value compares the control group to the ESPB group.

§ *P*-value compares the PENG group to the ESPB group.

∥ *P*-value compares all 3 groups.

ESPB indicates erector spinae plane block; FLACC, Face, Legs, Activity, Cry, Controllability Scale; PENG, pericapsular nerve group.

### Primary Outcomes

Lumbar ESPB and PENG block group patients had lower FLACC scores at all time points (30, 60, 90, and 120 min after surgery and 6, 24, and 48 h after surgery) compared with the control group. The PENG block group had lower pain scores 12 hours after surgery than the lumbar ESPB group (0.8±0.9 vs. 0.4±0.8, *P*=0.0278), as seen in Table 2.

**TABLE 2:** Primary Outcomes

	Control group (n=30)	PENG group (n=30)	ESPB group (n=30)	*P* ^†,^ ^‡^	*P* ^§,^ ^‡^	*P* ^‖,^ ^‡^	*P* ^¶,^ ^#^
FLACC score							
30 min after surgery	2.6 (1.4)	0.8 (1.1)	1.4 (1.1)	<0.0001*	<0.01*	0.13	<0.0001*
60 min after surgery	3.4 (0.9)	0.9 (1.0)	1.1 (1.0)	<0.0001*	<0.0001*	0.71	<0.0001*
90 min after surgery	3.7 (1.1)	1.2 (1.0)	0.6 (0.9)	<0.0001*	<0.0001*	0.08	<0.0001*
120 min after surgery	4.0 (1.3)	0.7 (0.9)	0.5 (0.9)	<0.0001*	<0.0001*	0.88	<0.0001*
6 h after surgery	3.1 (1.2)	0.8 (0.9)	0.5 (0.9)	<0.0001*	<0.0001*	0.41	<0.0001*
12 h after surgery	2.6 (0.8)	0.8 (0.9)	0.4 (0.8)	<0.0001*	<0.0001*	0.03*	<0.0001*
24 h after surgery	2.567 (0.8)	0.7 (0.9)	0.6 (0.8)	<0.0001*	<0.0001*	0.73	<0.0001*
48 h after surgery	2.5 (0.9)	0.3 (0.7)	0.3 (0.4)	<0.0001*	<0.0001*	0.93	<0.0001*

* Significant *P*-value.

† *P*-value compares the control group to the PENG group.

‡ Post hoc Tukey test used to compare means between the groups.

§ *P*-value compares the control group to the ESPB group.

‖ *P*-value compares the PENG group to the ESPB group.

¶ *P*-value compares all 3 groups.

# ANOVA test used to compare means between the groups.

ESPB indicates erector spinae plane block; FLACC, Face, Legs, Activity, Cry, Controllability Scale; PENG, pericapsular nerve group.

### Secondary Outcomes

Before the surgery, the NLR (*P*=0.3134) and PLR (*P*=0.7113) levels did not differ between the PENG block and control groups (*P*=0.1806), and the PENG block and ESPB groups (*P*=0.4743). However, NLR values significantly differed between ESPB and the control group before surgery with *P*=0.0111. Twenty-four hours after surgery, the NLR levels were significantly lower in both PENG (1.81±0.61) and ESPB (0.95± 0.50) compared with the control group (4.19±1.18), with both *P*<0.0001. However, the NLR levels between the PENG and lumbar ESPB groups did not significantly differ with *P*=0.7378.

The PLR levels before surgery did not significantly differ between the 3 groups. The PLR levels 24 hours after surgery were significantly lower in PENG (176.0±63.22) and lumbar ESPB (156.5±50.9) group, compared with the control group (318.9±98.3), with both *P*<0.0001. Also, the PLR level between the PENG and lumbar ESPB groups did not differ significantly with *P*=0.5618.

The total opioid consumption, within 24 hours after surgery, expressed in nalbuphine milligrams/kilogram was significantly lower in the PENG (0.09±0.10) and lumbar ESPB (0.06±0.08) compared with the control group (0.54±0.19), with *P*<0.0001. However, the total opioid consumption within 24 hours after surgery, expressed in nalbuphine milligrams/kilogram, did not significantly differ between the PENG and lumbar ESPB groups, with *P*=0.6925. Between 24 and 48 hours after surgery, the total opioid consumption expressed in nalbuphine milligram/kilogram was significantly lower in the PENG (0.00±0.00) and lumbar ESPB (0.00±0.00) compared with the control group (0.30±0.15), with *P*<0.0001.

All children in the control group required opioids for postoperative pain management, in contrast to the PENG (17 children) and lumbar ESPB (15 children) groups, with *P*<0.0001. The number of children who received opioids did not significantly differ between the PENG and lumbar ESPB groups, *P*=0.7961, as seen in Table 3.

**TABLE 3:** Secondary Outcomes

	Control group (n=30)	PENG block group (n=30)	ESPB group (n=30)	*P* †, ‡	*P* §, ‡	*P* ‖, ‡	*P* ¶, #
NLR							
Before surgery	1.37 (0.54)	1.12 (0.61)	0.95 (0.50)	0.18	0.01*	0.47	0.31
24 h after surgery	4.19 (1.18)	1.81 (0.94)	1.63 (0.56)	<0.0001*	<0.0001*	0.74	<0.0001*
PLR							
Before surgery	119.3 (41.5)	122.0 (48.5)	117 (39.6)	0.97	0.78	0.63	0.71
24 h after surgery	318.9 (98.3)	176.0 (63.22)	156.5 (50.9)	<0.0001*	<0.0001*	0.56	<0.0001*
Total opioid consumption expressed in nalbuphine milligram/kilogram							
Within 24 hours postoperatively	0.54 (0.19)	0.09 (0.10)	0.06 (0.08)	<0.0001*	<0.0001*	0.69	<0.0001*
Within 24-48 hours postoperatively	0.30 (0.15)	0	0	<0.0001*	<0.00001*	>0.9999	<0.0001*
OPIOIDS							
Yes	30	17	15	<0.0001*	<0.0001*	0.80	<0.0001*
No	0	13	15				

* Significant *P*-value.

† *P*-value compares the control group to the PENG group.

‡ ANOVA test with post hoc Tukey test used to compare means between the groups or Fisher exact test

§ *P*-value compares the control group to the ESPB group.

‖ *P*-value compares the PENG group to the ESPB group.

¶ *P*-value compares all 3 groups.

# ANOVA test used to compare means between the groups.

ESPB indicates erector spinae plane block; NLR, neutrophil-to-lymphocyte ratio; PENG, pericapsular nerve group; PLR, platelet-to-lymphocyte ratio.

No block-related complications were observed in the study.

## DISCUSSION

This double-blinded, randomized controlled trial (RCT) showed that ultrasound-guided PENG block and lumbar ESPB could improve children’s pain management, lower total opioid consumption, and lower stress response to surgery expressed by the NLR and PLR after a pediatric hip surgery. This is the first study comparing the effectiveness of the lumbar ESPB and PENG block in children’s hip surgery.

Due to limited data in the available literature regarding the effectiveness of PENG block and lumbar ESPB in pediatric hip surgery, we decided to compare those 2 regional blocks to no-block analgesia.

Our study showed that lumbar ESPB and PENG block reduced postoperative pain scores and cumulative opioid consumption. In our research, the FLACC pain scores were below 3 in patients receiving PENG block or ESPB at all time points after surgery. Also, almost half of the children receiving PENG block or lumbar ESPB did not require opioid medications for pain management, which contributed to improved functional recovery and reduced opioid-related adverse effects. It should be remembered that children undergoing hip surgery often require further surgical procedures. According to Philips et al,^21^ appropriate pain treatment and, above all, the absence of pain immediately after the procedure reduces perioperative stress and fear of another surgery. It shortens the patient’s stay in the hospital.

The pain scores in our study were significantly lower in the ESPB compared with the PENG block group 12 hours after surgery. This may suggest that ESPB provides more extended analgesia after pediatric hip surgery than PENG block. However, this conclusion was not confirmed by the opioid consumption in the first 24 hours. Until now, there have been no similar trials in the pediatric population. Our results are similar to those of Huda et al,^22^ who showed that lumbar ESPB in adult hip surgeries reduced postoperative opioid consumption and lowered pain scores up to 9 hours following surgery. Also, Abduallah et al^16^ showed that pediatric ESPB lowered pain scores and opioid consumption after surgery. Kim et al^23^ demonstrated that PENG block reduces postoperative opioid consumption and decreases pain scores in patients undergoing adult hip surgery up to 24 hours following surgery. Also, Yu et al^6^ showed that the PENG block in adults is an alternative multimodal analgesia to other regional blocks in hip surgery.

The PENG block and lumbar ESPB are generally considered safe and effective motor-sparing techniques. However, complications may include inadvertent vascular puncture, hematoma, or local anesthetic systemic toxicity, which remains rare in experienced hands.^6,18^ In addition, while these techniques avoid motor weakness, their effectiveness can depend on precise anatomic identification and local anesthetic spread, potentially leading to incomplete block efficacy.^24,25^ Importantly, in our study, no complications related to these blocks were observed in the pediatric patients undergoing the trial.

In this double-blinded, randomized controlled trial (RCT), the lumbar ESPB and PENG block reduced the stress response expressed by the PLR and NLR compared with the control group.

The ESPB was shown to lower the NLR and PLR levels in adults and children following spine surgery.^18,19^ It also reduced the IL-6 and IL-10 related to the stress response in adult posterior spinal fusion and decompression.^26^ Unfortunately, there are no similar trials regarding lumbar ESPB in children. Also, there are no comparable studies regarding the impact of the PENG block on surgery-induced stress response in adults or children.

Regional anesthesia probably influences the inflammatory and sympathetic response that occurs perioperatively due to vascular permeability, elevated blood flow, and leukocyte aggregation.^27,28^ The complete blood count estimates PLR and NLR from platelet, neutrophil, and lymphocyte values. The NLR and PLR are inflammatory signs that anticipate morbidity, mortality, and subclinical inflammation.^29^ Moosmann et al,^30^ in their recent study concerning children, showed that NLR levels were higher after birth with a decrease in the first 2 years of life. The NLR levels steadily rise from 3 to 18 years old, from 0.99 to 1.76 for both sexes. The NLR values >1.9^31^ and >1.97^32^ were shown to be associated with a higher incidence of developing sepsis in young children. Mathews et al^33^ showed that a rise in PLR >14 and a rise in NLR >2.0 was correlated with increased mortality among patients in pediatric intensive care units. The cutoff values to predict sepsis and mortality in children were determined by Pasaribu et al.^34^ They proved that the NLR cutoff value for septic patients was 3.52, with 82.50% sensitivity and 61.11% positive predictive value. The cutoff value for NLR as a predictor of mortality was 8.98, with 77.78% sensitivity and 58.3% positive predictive value. However, they did not find a correlation between the PLR values and sepsis or mortality.

The PLR values were found to continually increase from 63.36 in boys and 61.36 in girls in the first year of life to 118.8 in boys and 112.6 in girls at 18 years old.^30^ Shenoy and Patil^35^ showed the cutoff value for predicting mortality for NLR was 2.18, and for PLR, it was 34.1.

In our study, the NLR and PLR values were significantly lower in the ESPB and PENG block groups than in the control group. This might suggest that ESPB and PENG blocks block the afferent nociceptive stimulation of the injury site and enhance the result of intravenous pain management.

### Limitations

Our results must be interpreted cautiously, and several limitations should be considered. The main limitations of this study were the small sample size and the large volume of local anesthetic. We also did not obtain the NLR and PLR over 48 hours, assess the sensory block, review the duration of the block, or assess the adverse effects like nausea and vomiting, constipation, and itching, and we did not monitor the hospital discharge times. Further studies comparing ultrasound-guided PENG block and lumbar ESPB with the same long-acting local anesthetic are required to evaluate the best analgesic option in hip surgeries.

## CONCLUSIONS

On the basis of our findings, both ultrasound-guided ESPB at the L3 lumbar level and the PENG block are effective for postoperative analgesia. They can reduce pain scores and opioid consumption in children undergoing hip surgery. The lumbar ESPB and the PENG block lower the NLR and PLR levels, thus lowering the stress response following surgery. Therefore, we strongly recommend consideration of those techniques as a part of multimodal analgesia protocols in pediatric hip surgery.

## References

[1] Wu JP . Pediatric anesthesia concerns and management for orthopedic procedures. Pediatr Clin North Am. 2020;67:71–84. 31779838 10.1016/j.pcl.2019.09.006 PMC7172179

[2] Farrow L Faulkner A Gardner WT . Surgical management for developmental dysplasia of the hip. Orthop Trauma. 2020;34:324–331.

[3] Frawley G Marchesini V Loh B . Pediatric lower limb peripheral nerve blocks: Indications, effectiveness, and the incidence of adverse events. Pediatr Anesth. 2022;32:946–953. 10.1111/pan.14468 35451202

[4] Daroszewski P Huber J Kaczmarek K . Comparison of motor evoked potentials neuromonitoring following pre- and postoperative transcranial magnetic stimulation and intraoperative electrical stimulation in patients undergoing surgical correction of idiopathic scoliosis. J Clin Med. 2023;12:6312. 37834956 10.3390/jcm12196312 PMC10573895

[5] Yu L Shen X Liu H . The efficacy of pericapsular nerve group block for postoperative analgesia in patients undergoing hip surgery: a systematic review and meta-analysis of randomized controlled trials. Front Med. 2023;10:1084532. 10.3389/fmed.2023.1084532 PMC9998683 36910500

[6] Domagalska M Wieczorowska-Tobis K Reysner T . Pericapsular Nerves Group (PENG) block in children under five years of age for analgesia in surgery for hip dysplasia: case report. J Pers Med. 2023;13:454. 36983637 10.3390/jpm13030454 PMC10059249

[7] Kowalski G Leppert W Adamski M . Rectal enema of bupivacaine in cancer patients with tenesmus pain–case series. J Pain Res. 2019:1847–1854. 31354333 10.2147/JPR.S192308 PMC6578571

[8] Alhayyan A McSorley S Roxburgh C . The effect of anesthesia on the postoperative systemic inflammatory response in patients undergoing surgery: a systematic review and meta-analysis. Surg Open Sci. 2020;2:1–21. 10.1016/j.sopen.2019.06.001 PMC7391900 32754703

[9] Altinbaş A Bulut A . Mean platelet volume, neutrophil/lymphocyte ratio, platelet/lymphocyte ratio and early post-operative anesthesia complications. Turk J Biochem. 2023;48:403–409.

[10] Zahorec R . Neutrophil-to-lymphocyte ratio, past, present and future perspectives. Bratisl Lek Listy. 2021;122:474–488. 34161115 10.4149/BLL_2021_078

[11] Boruah P Gupta B Kumar A . Effects of different anesthetic techniques on neutrophil lymphocyte ratio and monocyte lymphocyte ratio in patients undergoing major non-cardiac surgery: a prospective, single-blind, randomized study. Bali J Anesthesiol. 2023;7:76–81.

[12] Tran J Agur A Peng P . Is pericapsular nerve group (PENG) block a true pericapsular block? Reg Anesth Pain Med. 2019;44:257. 10.1136/rapm-2018-100278 30635511

[13] Peng Philip W H Perlas Anahi Chin Ki Jinn . Reply to Dr Nielsen: Pericapsular Nerve Group (PENG) block for hip fracture. Reg Anesth Pain Med. 2019;44:415. 30777904 10.1136/rapm-2018-100234

[14] Domagalska M Ciftci B Reysner T . Pain management and functional recovery after pericapsular nerve group (PENG) block for total hip arthroplasty: a prospective, randomized, double-blinded clinical trial. J Clin Med. 2023;12:4931. 37568331 10.3390/jcm12154931 PMC10420102

[15] Tulgar S Kose HC Selvi O . Comparison of ultrasound-guided lumbar erector spinae plane block and transmuscular quadratus lumborum block for postoperative analgesia in hip and proximal femur surgery: a prospective randomized feasibility study. Anesth Essays Res. 2018;12:825–831. 30662115 10.4103/aer.AER_142_18 PMC6319070

[16] Abduallah MA Al-Ahwal LA Ahmed SA . Effect of erector spinae plane block on postoperative analgesia after pediatric hip surgery: randomized controlled study. Pain Pract. 2022;22:440–446. 35032350 10.1111/papr.13099

[17] Abotaleb AM Negm EE Abdelwahed WM . A comparative study of preoperative ultrasound-guided lumbar erector spine plane block and preoperative ultrasound-guided caudal block for postoperative pain control in pediatric lower limb surgeries: a randomized controlled trial. Egypt J Anaesth. 2023;39:802–809.

[18] Domagalska M Ciftsi B Janusz P . Effectiveness of the bilateral and bilevel erector spinae plane block (ESPB) in pediatric idiopathic scoliosis surgery: a randomized, double-blinded, controlled trial. Egypt J Anaesth. 2024;39:802–809. 10.1097/BPO.0000000000002707 PMC11232938 38689466

[19] Domagalska M Ciftsi B Janusz P . The neutrophil-to-lymphocyte ratio (NLR) and platelet-to-lymphocyte ratio (PLR) levels following erector spinae plane block (ESPB) in posterior lumbar decompression: a randomized, controlled trial. Eur Spine J. 2023;12:4192–4199. 10.1007/s00586-023-07913-z 37668689

[20] Flaviano E Bettinelli S Assandri M . Erector spinae plane versus fascia iliaca block after total hip arthroplasty: a randomized clinical trial comparing analgesic effectiveness and motor block. Korean J Anesthesiol. 2023;76:326–335. 36632641 10.4097/kja.22669 PMC10391077

[21] Phillips MR Adamson WT McLean SE . Implementation of a pediatric enhanced recovery pathway decreases opioid utilization and shortens time to full feeding. J Pediatr Surg. 2020;55:101–105. 31784102 10.1016/j.jpedsurg.2019.09.065

[22] Huda AU Ghafoor H . The use of erector spinae plane block reduces opioid consumption and pain score in postoperative period after hip surgery: a meta-analysis. Cureus. 2023;15:e47477. 38022340 10.7759/cureus.47477 PMC10662936

[23] Kim E Shin WC Lee SM . Efficacy of pericapsular nerve group block for pain reduction and opioid consumption after total hip arthroplasty: a meta-analysis of randomized controlled trials. Hip Pelvis. 2023;35:63. 37323546 10.5371/hp.2023.35.2.63 PMC10264226

[24] Reysner T Kowalski G Grochowicka M . The pericapsular nerve group (PENG) block for hip surgery. A narrative review. Chir Narządów Ruchu Ortop Pol. 2023;88:17–24.

[25] Domagalska M Reysner T . Pain management in total knee arthroplasty. A comprehensive review. Chir Narządów Ruchu Ortop Pol. 2022;87:173–180.

[26] Tantri AR Rahmi R Marsaban AHM . Comparison of postoperative IL-6 and IL-10 levels following Erector Spinae Plane Block (ESPB) and classical Thoracolumbar Interfascial Plane (TLIP) block in a posterior lumbar decompression and stabilization procedure: a randomized controlled trial. BMC Anesthesiol. 2023;23:1–8. 36624374 10.1186/s12871-023-01973-w PMC9830847

[27] Domagalska M Reysner T Kowalski G . Pain management, functional recovery, and stress response expressed by NLR and PLR after the iPACK block combined with adductor canal block for total knee arthroplasty—a prospective, randomised, double-blinded clinical trial. J Clin Med. 2023;12:7088. 38002702 10.3390/jcm12227088 PMC10672046

[28] Reysner M Reysner T Janusz P . Dexamethasone as a perineural adjuvant to a ropivacaine popliteal sciatic nerve block for pediatric foot surgery: a randomized, double-blind, placebo-controlled trial. Reg Anesth Pain Med. 2024;29:rapm-2024–105694. 10.1136/rapm-2024-105694 PMC12703320 39209730

[29] Duran H Alpdemir M Çeken N . Neutrophil/lymphocyte and platelet/lymphocyte ratios as a biomarker in postoperative wound infections. Turk J Biochem. 2022;47:756–762.

[30] Moosmann J Krusemark A Dittrich S . Age-and sex-specific pediatric reference intervals for neutrophil-to-lymphocyte ratio, lymphocyte-to-monocyte ratio, and platelet-to-lymphocyte ratio. Int J Lab Hematol. 2022;44:296–301. 34816610 10.1111/ijlh.13768

[31] Bekdaş M Göksügür SB . Neutrophil/lymphocyte and C-reactive protein/mean platelet volume ratios in differentiating between viral and bacterial pneumonias and diagnosing early complications in children. Saudi Med J. 2014;35:442–447. 24825803

[32] Dursun A Ozsoylu S Akyildiz BN . Neutrophil-to-lymphocyte ratio and mean platelet volume can be useful markers to predict sepsis in children. Pak J Med Sci. 2018;34:918. 30190753 10.12669/pjms.344.14547 PMC6115542

[33] Mathews S Rajan A Soans ST . Prognostic value of rise in neutrophil to lymphocyte ratio (NLR) and platelet to lymphocyte ratio (PLR) in predicting the mortality in paediatric intensive care. Int J Contemp Pediatr. 2019;6:1052–1058.

[34] Pasaribu FM Setyaningtyas A Andarsini MR . Neutrophil to lymphocyte ratio, monocyte to lymphocyte ratio, platelet to lymphocyte ratio, mean platelet volume as a predictor of sepsis mortality in children at Dr. Soetomo General Hospital. Crit Care Shock. 2021;24. https://openurl.ebsco.com/results?sid=ebsco:ocu:record&bquery=IS+1410-7767+AND+VI+24+AND+IP+2+AND+DT+2021&link_origin=scholar.google.com

[35] Shenoy S Patil S . Neutrophil lymphocyte ratio and platelet lymphocyte ratio as predictors of disease severity and mortality in critically ill children: a retrospective cohort study. J Pediatr Intensive Care. 2023. doi:10.1055/s-0043-1768661.

